# Exploring the feasibility of distributed ledger technology for tamper-proof logging in MANETs: challenges and solutions

**DOI:** 10.1038/s41598-026-52494-8

**Published:** 2026-05-14

**Authors:** Nirmalkumar S. Benni, G. C. Jagan, C. Tamizhselvan, Manjunath G. Asuti

**Affiliations:** 1 Department of CSE (AI & ML), RNS Institute of Technology, Bengaluru, 560098 India; 2 Department of Electronics and Communication Engineering, Jeppiaar Engineering College, Chennai, 600119 India; 3 Department of Data Science and Business Systems, School of Computing, SRM Institute of Science and Technology, Kattankulathur, Chengalpattu District, Tamilnadu 603203 India; 4 Department of Electronics and Communication Engineering, B N M Institute of Technology, Bengaluru, Karnataka 560070 India

**Keywords:** Blockchain, Distributed ledger technology (DLT), Cyber-physical systems (CPS), Energy science and technology, Engineering, Mathematics and computing

## Abstract

Mobile Ad Hoc Networks, also known as MANETs, are complex Cyber-Physical Systems which require a reliable and secure infrastructure due to the enormous amounts of data that are generated by users. MANETs are a collective term that refers to all Internet-enabled gadgets, sensors, and actuators, regardless of whether or not each of these components is linked to mobile networks. There is the potential for even a single high-tech mobile device to generate enormous volumes of data. During the course of this research, Distributed Ledger Technology is investigated, and information is gathered on consensus procedures and the possible applications of these techniques in MANETs, which are the basis of wireless networks. At the moment, there are a number of distributed ledger networks that are operational. There are a variety of decentralized applications; some are more often seen in the financial sector or supply chains. The blockchain may be decentralized and safe, but there are costs and benefits to using any of these networks. A consensus method that meets the needs of MANETs may be designed using the results of this investigation.

## Introduction

Due to the absence of trust and reputation between the nodes involved in the routing process, Mobile Ad hoc Networks (MANETs) often experience unreliable packet delivery^1^. There is a severe lack of power for nodes in MANETs due to their reliance on batteries. That is why one of the most significant optimization criteria for MANETs is energy efficient routing. Nodes are exhausted and the network is partitioned prematurely because traditional routing techniques do not take node energy into account when choosing routes^2^.The MANETs, or mobile ad hoc networks, have begun to rely on wireless connections. While research indicates that hackers efficiently launch assaults at the network layer, the dynamic nature of MANETs makes them vulnerable to critical attacks at the OSI levels^3^. No matter whether the network is wireless or wired, routing remains a significant difficulty. The routing problem is particularly acute in MANETs, which are wireless networks that are both decentralized and highly adaptable. Malicious nodes in a MANET may also hinder the network’s routing capabilities^4^. In situations when a fixed network is not available, a new paradigm for dynamic communication among mobile nodes has arisen: mobile ad hoc networks, or MANETs. On the other hand, malicious nodes may disrupt communication and disrupt network operations by taking advantage of MANETs’ inherent decentralization and self-organization^5^. As they travel from one location to another, the nodes that make up a MANET interact with one another using wireless networks. Due to its infrastructure-less nature and topology-changeability, MANET is highly attackable. Preventing attacks on MANETs is a major challenge. A network-based assault originates from a malicious node in the network. Multiple types of assaults may compromise a MANET, each of which can affect the network’s functionality differently^6^. More and more low-end routers might pose security risks as the number of Internet of Things devices continues to skyrocket. When routers aren’t performing properly, they might refuse data forwarding services and selectively discard packets, reducing network availability. There is a danger of one-point failure due to the centralized design of the RM center, even if current RM systems are helpful in spotting problematic routers^7^. One generous strategy for sending packets to the BS in a MANET is known as MANET routing. Malicious nodes may complicate mobile ad hoc network activities while routing. This is why a reliable distributed protocol for routing is necessary to keep mobile ad hoc networks efficient and robust in their routing support^8^. Since the late 1970s, researchers in the age of distributed systems have poured a lot of time and energy into studying the fault-tolerant consensus issue. The advent of blockchain technology has brought it back into the spotlight. Scalability and efficiency, however, continue to be points of contention within the agreement. Partitioning networks into many committees using sharding methods is one such option^9^. Various tangible items may be used in today’s technology setting to ease human labor. A state-of-the-art technical solution, the IoT links physical items to the digital realm via various communication and network technologies. By integrating with the IoT in smart settings, MANET increase the device’s economic feasibility and user appeal. By combining wireless sensors with MANET, a new system based on the Internet of Things and IT-network might be developed^10^. The ever-changing nature of MANETs makes achieving secure communication a significant task. Several researches on security have shown that reputational management systems may be effective with minimal effort. Automated evaluation techniques based on a preset trust scheme determine a node’s reputation.^11^. It is possible for mobile nodes to connect with one another in a MANET even in the absence of fixed connection points or base stations since the network’s architecture is not preset or established. A node in a MANET may serve dual purposes, being both a host and a router. Intermediary nodes allow packet transmission and reception between nodes in the network. On the other hand, MANET performance is drastically reduced when selfish and malicious nodes are present^12^. Connected wirelessly, mobile nodes in a MANET exchange data packets with one another autonomously, without the need for a governing body or centralized infrastructure. Due to its dynamic topologies and limited resources, providing a power-aware safe routing algorithm is a formidable challenge in such a network^13^. One definition of a MANET is an independent system of nodes; another is a decentralized wireless network of mobile devices. The whole network is comprised of mobile nodes that are linked together by wireless connections. Without the need for preexisting network infrastructure, they may join forces to create a network. A relatively new area of research, MANET makes use of blockchain technology in a wireless ad hoc setting^14^. When a wired network goes down, users may fall back on infrastructure-less MANETs to keep the conversation going. Attack detection in MANETs relies heavily on gathering security-related data. In order to collect information, a node has to identify routes to other nodes that are accessible, and in order to choose routes that are safe, it needs to obtain information on security during route discovery^15^. Modern MANET technology has several potential uses, including in the military, in civilian spheres, and in catastrophe recovery networks. Assuming all nodes are honest and cooperative is the operating principle of MANET routing algorithms. Such an assumption exposes MANET to more manipulation and interception, which can be used to commit a host of questionable attacks, including Denial of Service attacks^16^.To create a self-organizing system that doesn’t depend on fixed infrastructure, save for gateways to other networks, the nodes in MANETs serve as both hosts and routers. With the help of trust computation, MANETs may make their nodes work together more effectively, which is crucial for security reasons^17^.

### Objectives

- *Decentralization* because nodes in MANETs may join and leave the network at will, trust assessment for resident nodes is a crucial part of this network architecture. But evaluating nodes that are more than one hop away from a single hub is obviously not feasible. Thus, MANETs rely heavily on decentralized trust evaluation, which means that MANET nodes should engage in simple, decentralized mutual assessment.
- *Availability & consistency* One crucial consideration for decentralized systems like MANETs is data availability. Even when they are located at a multi-hop distance, each node should still have confidence levels for the relay nodes it uses to transmit data. Furthermore, every node should have consistent access to the data.
- *Tamper-proofing* every node in the network will have a link to all the available information if the design goals of decentralization and availability are met. Critical information should remain untouchable by malevolent nodes in the network, even with dependable backing from the suggested approach. So, it’s important that the system can withstand attacks from a limited group of coordinated enemies.
- *Lightweight performance* given the gravity of the security concerns, it is imperative that any trust solution, notwithstanding the complexity of the authentication calculations, provide respectable speed. Instead of using a complex mechanism that is highly secure, a simple and effective approach would be better for MANETs with minimal resources.
- Efficiency of security model in MANETs: The ever-changing nature of MANETs seems to need the execution of repeating procedures in the majority of trust solutions. When nodes in a network don’t operate together, the system is more likely to be attacked several times by the same malicious actor. Thus, it is necessary to provide a more efficient trust solution for MANETs.

## Preliminaries

### Distributed ledger technology

A distributed ledger is a database that is accessible from several nodes in a network, regardless of their physical location. A distributed ledger is one that is spread out and maintained on a worldwide scale; it is a compilation of financial accounts. A number of entities located in various places and with varying levels of authority hold and rearrange distributed ledgers. No central authority is required for the database entries in distributed ledgers. No one else can change the records once they are added to distributed ledgers. They can’t change the records until the ledgers are handed out.

Many different technologies fall under the umbrella name "distributed ledger technology," or DLT, which allows for the transparent, decentralized, and secure storing of data across a system of users. To explain DLT from the top down, you have to break it down into its component parts and then build on top of that. A detailed description of DLT may be found here. Consensus is a way to keep the records consistent. The data placed into the shape database is supposedly what was meant to be there, according to everyone. Hashing, a cryptographic technique, is relied upon since memory locations cannot be utilized for linking. By using cryptographic algorithms, consensus, and replication, blockchains ensure that the records’ integrity is protected via hash linking. Consequently, a blockchain is an immutable record system that is distributed, replicated, and managed to keep data intact. Another school of thought maintains that blockchain records transactions and events in an immutable ledger that cannot be tampered with, either intentionally or unintentionally. It is almost hard to change the records. Banking institutions employ the blockchain technology to standardize human activities in a decentralised setting. We want to protect the privacy of our clients as well as the secrecy of their transaction records in a decentralized network. It is the financial institution that receives the settlement from the payer via blockchain technology, and it is organizing financial institution that receives it thereafter. A letter of encouragement, requesting confirmation, is thereafter sent to the payee by the coordinating financial institution. Currently, the archive is provided to the financial organization managing the arrangement once the payee submits it. A creative settlement between the payer and the payee may be initiated with the help of this archive, which the payer obtains. By doing so, a blockchain-based covered trade between persons may be carried out.

**Fig. 1:**
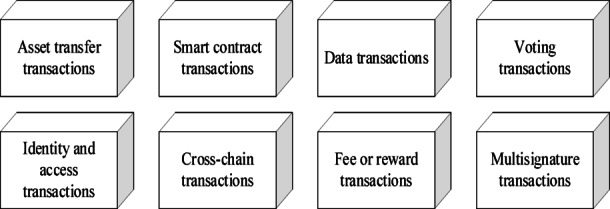
Transaction types.

**Fig. 2:**
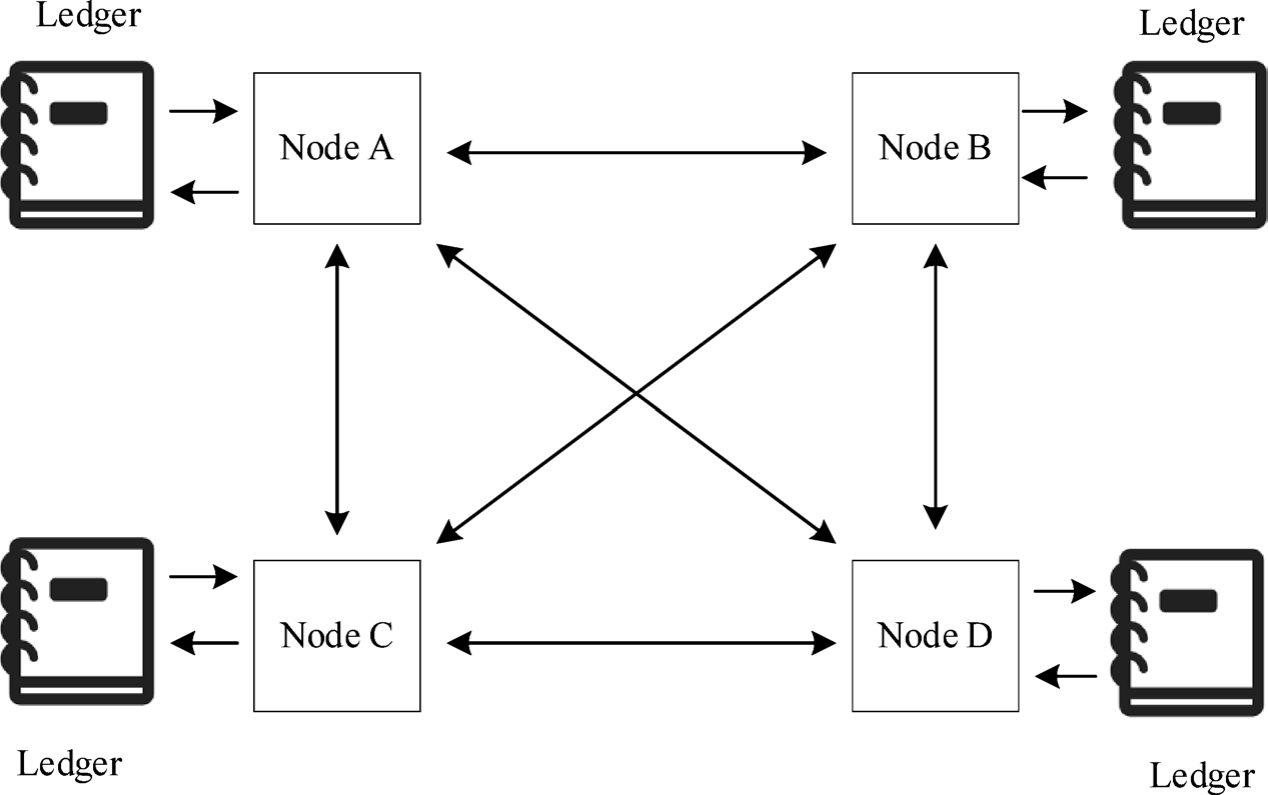
Distributed ledger in MANETs.

A transaction in distributed ledger technology (DLT) is any action or occurrence that changes or transfers digital assets, information, or other network resources. Figure 2 shows the basic building blocks of DLT systems, which are transactions. These transactions record and maintain an overview of assets and transactions between members in the network. Transaction Types are in Fig. 1.

- Ledger structure
  A ledger is an information structure that documents transactions and provides a verifiable record of all network activity in a distributed ledger technologies (DLT). In distributed ledger technology, a linear blockchain, such as in Bitcoin and Ethereum, or a Directed Acyclic Graph (DAG), such as in IOTA is defined by the specific implementation.
- Consensus algorithms
  Digital ledger technology relies on consensus algorithms to ensure the integrity of the ledger by facilitating agreement amongst network nodes on the veracity of transactions. Two popular methods for reaching a consensus are Proof-of-Work (PoW) and Byzantine Fault Tolerance (BFT). When it comes to efficiency, safety, and resource consumption, every mechanism has its own set of pros and cons. At its heart, the technology rests the consensus algorithm, which underpins decentralization, boosts safety and openness, and plays a pivotal role in latency and throughput on networks.
- Cryptography
  To guarantee confidentiality, authenticity, and integrity of data, DLTs use a number of cryptographic procedures and methods. Among the most important methods and processes are:
  Public key cryptography (PKC): an example of asymmetric cryptography is the use of a public key and a private key in PKC. The blockchain technology employs a type of address to users (public key), and a type of transaction and asset transfer authorization (private key). RSA and ECC are a couple of examples of public key cryptography (PKC) algorithms that are applied to distributed ledger technology (DLT).
  Cryptographic key derivation functions (KDFs): KDFs are employed to generate cryptography keys, such as passwords or passphrases, based on user-provided inputs. It becomes more difficult for attackers to guess or brute-force keys in DLT due to KDFs, which boost key security. The DLT uses key distribution functions (KDFs) such as scrypt, bcrypt, and Argon2.
  Cryptographic consensus mechanisms: consensus procedures rely on cryptography to keep the distributed ledger secure and uncompromised. There are a number of examples of proof-of-work (PoW) and proof-of-stake (PoS) systems that use cryptographic problems to verify transactions and generate new blocks, respectively.
- Hash functions
  These mathematical methods accept an input and generate an output of a defined size; this result is known as a hash. Data ledger technology relies on hash functions for creating unique identities, ensuring data integrity, and making it resistant to tampering. Hash functions utilized in DLT include some such as SHA-256, which is used by Bitcoin, and Keccak-256, which is used by Ethereum.
- Consensus algorithms digital signatures
  With digital signatures, the sender may verify the message’s legitimacy and integrity by signing it using their private key. Digital signatures play a crucial role in DLT, allowing for the authorization of transactions and the verification of ownership. The Edwards-curve Digital Signature Protocol (ECDSA) and ECDSA are two popular digital signature algorithms in distributed ledger technology (DLT).
- Privacy-enhancing techniques

To further ensure anonymity in DLT, cryptographic methods are used. Below are a few instances:

- ZKPs allow users to prove the validity of an announcement without revealing any more information. The zk-SNARK and zk-STARK ZKP systems are examples of DLT implementations.
- Using these methods, sensitive information, such as the value of a transaction, may be kept secret. Bulletproofs and Pedersen commitments are two examples; the latter is used in protocols built on Mimblewimble and Monero.
- Ring signatures: These signatures make a transaction seem like it came from someone in the identical group as the receiver, making it impossible to tell who sent it. One implementation of ring signatures in DLT is CryptoNote, which is utilized in Monero.

Data integrity, privacy, and security in DLT systems are built upon these cryptographic procedures and methods. To meet new problems and improve DLT’s capabilities, new cryptographic approaches may be created and used as the technology develops (Fig. 2).

## Related work

Most of the academics in the^18^ survey found that MANETs are vulnerable to attacks at the network layer, and that article details such attacks. Packets are often sent to their destinations via AODV protocol routing and other protocols. Log files store information about transmitted packets; MANETs utilize data extraction, support vector machines, genetic programming, and other machine-learning methods to monitor the path of packets from these log files. In addition, the methods and approaches that have been suggested for identifying and forecasting these assaults from different types of incursions inside MANETS are covered. Reputation opportunistic routing using Q-learning (RORQ) is a novel reinforcement learning routing technique suggested for MANETs in^19^. A reputation system may identify and block harmful nodes in a network to ensure optimal routing by using that game-theory based protocol. As a result, their technology is able to locate a routing route in a hostile environment with more effectiveness. The simulation results showed that the proposed strategy has a chance to beat other state-of-the-art routing algorithms. Regarding energy economy, average end-to-end delay, and packet loss, the proposed method surpassed competing algorithms in both the black hole and gray hole attack scenarios. An analysis of Reputation-Based Opportunistic routing algorithms is presented in the review article^20^. RORPs use Q-Learning techniques to fortify MANETs against attacks by malevolent nodes. Authors examine present-day RORPs in detail, looking at their fundamental principles, inner workings, and comparative performance reviews. An in-depth overview of the key ideas, problems, and security threats related to MANETs is provided first. Then, they go into reputation-based routing ideas, highlighting the importance of reputation administration when distinguishing between legitimate and malicious nodes. Afterwards, the paper delves deeper into how Q-Learning techniques may be integrated into RORPs. It highlights how reinforcement learning methods can adjust routing choices based on the evolving network state as well as the dynamic reputations of nodes that are involved. That study delves into the possibilities of computer-assisted solutions for MANET and covers 38 research articles of opportunistic routing protocol. Their next step is to classify the RORPs that were part of the study according to how they handle cooperative enforcement, learning algorithms, and reputation computation. The many types of attacks, their effects on MANET, with the present defense methods based on MANET are detailed in the article^21^. The proposed SRA, SRABC, uses blockchain technology to verify nodes and prevent assaults on MANET. To protect control and data flow from potential dangers, blockchain technology has suggested the secure routing method. To do that, a Hash Function is generated for each and every transaction. The safety of the MANET will be their first topic of discussion. Part two of that paper delves into blockchain’s function in MANET safety. The SRA is defined with relation to blockchain technology in Sect.  3. The fourth step is to use PDR and Throughput to do an SRA evaluation on the Blockchain. The authors of^22^ propose a reputation management platform based on blockchain for Internet of Things networks as a way to circumvent the shortcomings of centralized routers RM systems. The proposed solution is a distributed database that employs blockchain technology to record router reports used in reputation calculation. An evaluation of each router’s dependability and the isolation of malevolent, misbehaving routers with poor reputations would be achieved by using the suggested reputation calculation technique. Beyond that, they enhance the effectiveness of block production while decreasing resource consumption by developing an optimal group mining procedure for the blockchain approach. To tackle these challenging issues, the research suggests a Secured Dynamic Optimal Router in MANET using a blockchain-based Auto-Metric Graph Neural Network^23^. They imitate the proposed procedure using the NS-2 program. In comparison to other models like the BC-SDOR-MANET-E-BATMAN and the BC-SDOR-MANET-GAHC, which use BC technology in MANET to improve ad hoc on-demand routing distance vector security, the suggested BC-SDOR-MANET-AGNN approach achieves minimal delays of 76.26%, 65.57%, and 42.9% in a 25% malicious routing surroundings, and less delays of 73.06%, 63.82%, and 38.84% in a 50% malicious routing environment. An improved method for hierarchical clustering, the divide-well-merge algorithm, was used in the^24^ article. The two halves of that process are the “Dividing” and “Merging” stages. The Advanced Ad-hoc On-demand Distance Vector Protocols is a tool for MANET route identification and maintenance that uses the Fire Hawk Optimization Algorithm to find the best possible multipath based on factors like distance, energy, trust, throughput, node degree, bandwidth, and node connectivity. That protocol can handle a large number of terminals without any loops and is very scalable. To improve secure data transfer and decrease packet loss, that approach establishes route discovery as well as route maintenance. For better data transfer security, the TriChain modified blockchain is suggested. For the purpose of MANET-IoT security, they presented their novel Directed Acyclic Graph-Blockchain design in^25^. Securing the network is the job of the MF-PUF authentication method. Every allowed node on the network is part of a cluster. To guarantee reliable data transmission, they set up the Jelly Fish Optimization approach after meticulously evaluating several elements. For comprehensive packet inspection, the authors recommend a Fully Linked Recurrent Neural Network. A system that uses deep packet inspection finds the incursions and blocks them. By using a combined algorithm, the suggested method improved upon previous approaches in terms of PDR (Packet Delivery Proportion), production, time evaluation, detection accuracy, and security levels. The results of the comparison show that the proposed study is better than all of the previous research in several respects. Node Activity-based Trust and Reputation estimate are two approaches proposed in the study for MANET safety and quality of service routing in^26^. Finding out how reliable and trustworthy a node is is what NA-TRE is all about. By monitoring shifts in activity, forwarding of packets, and dropping, the NA-TRE approach may be used to ascertain a node’s condition. Nodes are monitored for their many statuses, including Normal, Resource Constraints, and Malicious. That condition of the node is helpful for figuring out reputation and trust. The effectiveness of MANET is assessed in that study by comparing the NA-TRE method to preexisting protocols such as AODV, FACE, and TMS. Results from the experiment demonstrate a 20% improvement in throughput, a 10% reduction in cost, and a 0% increase in end-to-end latency. To identify selfish nodes and compel cooperation, a simple reputation-based system is suggested in^27^ that makes use of consumption and donation data. Disconnecting uncooperative nodes frees up resources for trustworthy nodes in the network. In the face of both selfish and malevolent assaults, their suggested system achieves better performance in terms of packet delivery fraction, normalized routing load, and packet loss than the benchmark scheme, according to the simulation findings. In addition, when contrasted with the benchmark technique, their method efficiently and precisely finds the selfish nodes. The study presents a reputation-based, power-aware safe routing mechanism in^28^. In that work, a safe route from the beginning node to the destination is established using the Krill Herd oriented Grasshopper Optimization Algorithm, a reputation model, and power. Node mobility, node actual capabilities, prior records, as well as reputation among neighbors are all reputation factors included in the reputation model. Establishing a safe route from the originating node to the destination takes into account the reputation elements for each node and the suggested KH-GOA method, which is a combination of Krill Herd with Grasshopper Optimization method. Reputation, power, distance, and latency are some of the multi-objective functions used by the suggested KH-GOA based routing protocol. The suggested KH-GOA outperforms the state-of-the-art in terms of power (27.313W), throughput (0.670), latency (0.114 ms), and detection rate (0.734 ms). An ensemble algorithm-based blockchain MANET is suggested in the paper^29^. Byzantine Fault Tolerance protocol-based blockchains are the backbone of the proposed scheme’s distributed MANETS routing environment. Implementing blockchain technology into MANET as an approach protocol via the use of BATMAN, a more efficient mobile ad hoc networking approach. The Extended-BATMAN method incorporates the blockchain concept into the BATMAN system using MANET. By enforcing many security procedures on each node separately, blockchain—a trustworthy, autonomous, and secure platform—eliminates most of the security issues around BFT. Four parameters—energy, packet delivery stages, average end-to-end latency, and network throughput—are empirically investigated using the recommended ensemble approach. The study^30^ introduces the Jaya Cuckoo Search algorithm, an optimization method, to address the security challenges. Initiating a safe route between the MANET nodes, this method merges the Jaya and Cuckoo Search algorithms to guarantee that the path achieved is both feasible and safe. The proposed JCS algorithm selects a secure path by means of a fitness function that considers a multi-objective function with considerations for energy, trust, distance, connection lifetime, latency, and reputation. At energy, throughput, as well as PDR values of 27,826, 0.554, as well as 0.628, respectively, the JCS algorithm demonstrates its peak performance^31^. Here are the procedures outlined in the proposed work: One, deploying guard nodes around the network and implementing zone-wise authentication using the BLAKE-3 hashing method. (2). The Rewards Optimized Deep Q Learning algorithm’s job is to do environmentally conscious clustering. (3). The PSO approach introduces a secure priority-based routing system. Here, they use the bi-fitness optimizer. Lastly, (4) SALSA 20 is encrypted using the Sensitivity Aware data encryption method, which evaluates the sensitivity level of a packet. To run the simulation, authors use the NS3.26 network simulator, which measures performance according to quality of service indicators. The research team behind the proposed OLSR procedure in^32–38^ used a deep-learning model to come up with a new approach. The Kaggle dataset is first used to gather the input videos. To locate the black-hole node, we will next use a novel model according to twin attention, SA DCBiGNet. After analyzing the trust values of the adjacent nodes, the improved osprey-aided optimum linked state routing protocol technique is used to complete the routing process. The EOOA, which stands for extended osprey optimization algorithm, also uses metrics like node and connection stability to choose the best feature. The integration of blockchain storage, which makes use of IPFS technology, enhances the privacy of MANET data. Furthermore, a delegated proof-of-stake consensus method is used to verify the proposed blockchain system.

Recent studies on trust computation in MANETs primarily rely on reputation-based, reinforcement-learning-based, or behavior-monitoring approaches to identify selfish and malicious nodes. While these mechanisms improve routing reliability, most existing trust models depend on either local observations or centralized reputation managers, which makes them vulnerable to false reporting, single-point failures, and coordinated attacks. In highly dynamic MANET environments, maintaining consistent and tamper-proof trust information across nodes remains a significant challenge.

Blockchain and Distributed Ledger Technology (DLT) have recently emerged as promising solutions for decentralized trust management due to their immutable, transparent, and distributed nature. Unlike conventional trust frameworks, blockchain-based trust systems eliminate the need for centralized authorities by allowing nodes to collectively validate and record trust-related events in a tamper-resistant ledger. Several recent works have explored blockchain-enabled MANET security, focusing on secure routing, reputation sharing, and intrusion detection. For instance, blockchain-assisted routing schemes have been proposed to mitigate packet-dropping attacks by recording forwarding behavior in an immutable ledger, while consensus mechanisms ensure agreement among nodes even in the presence of adversaries.

However, most existing blockchain-based MANET solutions suffer from high computational overhead, excessive latency, and scalability limitations, making them unsuitable for resource-constrained mobile nodes. Furthermore, limited attention has been given to integrating lightweight consensus mechanisms and adaptive routing cost functions that jointly consider node reputation and path efficiency. Motivated by these limitations, this work explores the feasibility of Distributed Ledger Technology for tamper-proof logging in MANETs and proposes a lightweight blockchain-assisted routing framework using SBO-CNN, where node reputation is securely recorded on-chain and dynamically incorporated into routing decisions.

## Proposed work

### Network model

In a MANET, the area of interest is divided into *K* finite grids denoted by $k\in \{1,\dots ,K\}$, as shown in Fig. 1. Even though Fig. 1 shows equal sized grids, our approach supports any cell shape and size. Let $N=\left\{{n}_{1},\dots ,{n}_{N}\right\}$ be the set of *N* wireless nodes and $M=\left\{{m}_{1},\dots ,{m}_{M}\right\}$ be the set of *M* miners in a given area. The nodes and the miners are mobile entities with omnidirectional antennas having fixed transmission range. Distributed nodes can interchange roles as routing Node or Miner. Typically, nodes with more processing power are likely to assume the role of a Miner. Thus, there are typically more nodes than miners *(M≪ N)* in any given grid. The nodes may route data packets to any other node within the same or different grid, as shown by red line in Fig. 1.

- Phase Thorough Analysis In order to identify the security flaws in MANETs, a comprehensive review of the available models was conducted. The process of regulating the trust between mobile nodes whilst broadcasting and receiving information from additional nodes was thoroughly examined.
- Phase Constructing the Model During this stage, we implemented a globally distributed reputation system to lessen the aforementioned risk and concentrate on preventing the spread of misinformation in MANETs caused by malevolent nodes.
- Phase 3: Putting the Model to the Test Based on the concept, a prototype was constructed and put through its paces in virtual testing. Due to the research-oriented nature of this work, the blockchain architecture prototype was tested and validated using data collected from simulated scenarios. The data was created using the truffle framework and the ganache blockchain emulator.

### Threat model

1. Malicious Nodes (Routing threat): An adversarial node may launch a black hole attack by spreading misleading routing information and swallowing all incoming traffic. Grey hole attacks include the selective dropping of packets; for instance, all data packets are discarded but routing packets are transmitted. By changing the target address or sending packets to the incorrect next hop node, an unauthorized node might cause data packets to be sent to the wrong place in a misrouting attack. We demonstrate that the aforementioned dangers are reduced when nodes are given reputations according to their past forwarding activities.
2. Malicious Miners (Blockchain threat): The block that is mined by malicious miners can include fabricated transactions. Traditional blockchain dangers, such as a 51% assault, do exist, yet they decrease in the degree of probability with the extension of the chain, with the increase in the number of packets being routed, and with the number of miners in the network. Forging the most recent or current blocks is the consequence of an attack that succeeds. With the possibility of one day assuming the function of a node and packet routing dependent on the reputation of intermediary nodes, miners have an inherent incentive not to lie. Over time, we give reputations to nodes based on the activities recorded in the blockchain. Then, we utilize nodes’ reputations to determine the shortest, most reputable route for packets to take.
3. Blockchain technology is used to store smart contracts. Which means you can’t change its data or instructions in any way. Smart contracts can not be changed after they have been implemented. Each time the system is updated, the most recent state of the smart contract is incorporated in a newly mined block and broadcasted to all participating nodes throughout the world. Instead than storing the blockchain locally, the mobile nodes in MANETs interact with it. Contributing nodes in the blockchain may connect with the mobile nodes, and the mobile nodes can get a copy of the blockchain. There are three primary purposes served by the smart contract.
  A method for adding mobile nodes to the blockchain
  A method for changing a node’s trust factor
  A method for getting the node’s trust factor back

Mobile nodes are able to interface directly with these three services, and they manage the reputation model’s key procedures. The most recent block on the blockchain includes the nodes’ most recent trust factors.

In addition to conventional black hole and gray hole attacks, the integration of Distributed Ledger Technology into MANETs introduces new attack surfaces that must be addressed. One such threat is the False Reputation Injection Attack, where compromised nodes attempt to submit fabricated forwarding records to artificially inflate or deflate reputation scores stored on the blockchain. Although blockchain immutability prevents direct modification of past records, malicious miners may collude to include falsified transactions in newly mined blocks.

Another emerging threat is the Consensus Manipulation Attack, in which adversarial miners attempt to influence block validation by controlling a subset of mining nodes within a grid. This attack aims to prioritize forged blocks or delay honest block propagation, potentially affecting trust computation temporarily. Additionally, Ledger Forking Attacks may occur during network partitions, where multiple ledger versions are created and later merged, leading to inconsistencies in reputation data if not handled properly.

Finally, Resource Exhaustion Attacks target blockchain-enabled MANETs by flooding the network with excessive trust update transactions, thereby increasing mining overhead, energy consumption, and latency. These attacks are particularly critical in MANET environments due to limited battery power and computational resources. The proposed blockchain-based reputation framework mitigates these threats by leveraging distributed consensus, historical reputation aggregation, and miner incentives that discourage dishonest behavior.

Let ${B}_{l}$ signify the ${l}^{th}$ transaction on a blockchain with L nodes. For block B_l, the forwarding rating of node n_i is represented by ${S}_{i}^{l}\in [-\mathrm{1,1}]$, represents the net forwarding behavior of node n_i is recorded in block ${B}_{l}$, including both its positive and poor activities,

1 $$S_{i}^{l} = \frac{{{\mid }~goodactions~E|_{i}^{l} - {\mid }~badactions~E|_{i}^{l} }}{{{\mid }~goodactions~E|_{i}^{l} + {\mid }~badactions~E|_{i}^{l} }}$$

where $|\cdot {|}_{i}^{l}$ stands for the count of the specific operation carried out by the node ${n}_{i}$ as recorded in ${B}_{l}$. The value of *∣* upright actions ${E|}_{i}^{l}$ (or *∣* corrupt actions ${E|}_{i}^{l}$ ) is calculated by tally of all transactions in block ${B}_{l}$. that pertain to node ${\eta}_{i}$(i.e., where the node id field corresponds to ${\eta}_{i}$ IP address) and have a flag of 1 (or 0).

### Reputation metric

Using the data recorded on the blockchain, we can determine ${\eta}_{i}$ reputation. The non-linear sigmoid function defines the reputation, R_i ∈ [0,1], for a blockchain of length L, and its exponent. ${\eta}_{i}$ represents the average of the forwarding ratings of the node n_i for every block and the historical weighted average of the block’s difficulty, represents the average of the forwarding ratings of the node n_i for every block and the historical weighted average of the block’s difficulty, ${D^l}S_i^l$,

2 $$R_{i} = \frac{1}{{1 + e^{{ - \eta_{i} }} }}, where, \eta_{i} = \frac{\beta }{{L \cdot D_{\max } }}\left( {\mathop \sum \limits_{l = 1}^{L} D^{l} S_{i}^{l} } \right)$$

When the exponent, ŷ_i, is mostly positive (or negative), the sigmoid reputation function, R_i, asymptotically approaches a value of 1 (or 0), with β = 8 being the sensitivity factor that causes this to happen. Nodes that are regularly excellent or appalling will ultimately get to a reputation of 1 or 0, respectively, in the long run, based on the nonlinear reputation function. On the other hand, nodes whose reputation is very sensitive to changes in behavior will see large swings in that measure. The reputational routing method is based on the reputation value that is generated in (3). Such a value provides a worldwide view of the honest behaviour of any node as trailed by miners over time.When node n_i sends an RREQ packet to its neighbor $n_{i + 1}$, the cost of the link (for the connection between $n_{i}$ and $n_{i + 1}$ ) is intended through $n_{i + 1}$ and is given by,

3 $$c\left( {n_{i} ,n_{i + 1} } \right) = \alpha + \left( {1 - \alpha } \right)\left( {1 - R_{i} } \right)$$

The node $n_{i + 1}$ raises the RREQ packet′s route cost field using $c\left( {n_{i} ,n_{i + 1} } \right)$. T The cost related to the length of the connection, or the hop-count, is the first term in (4). For a single link, the hop-count is 1.The second term is the costs incurred due to the goodwill of the node which sent the RREQ packet.When a node’s reputation is high, the reputation cost is near to 1, and vice versa, according to the phrase (1-R_i ┤).

**Fig.3:**
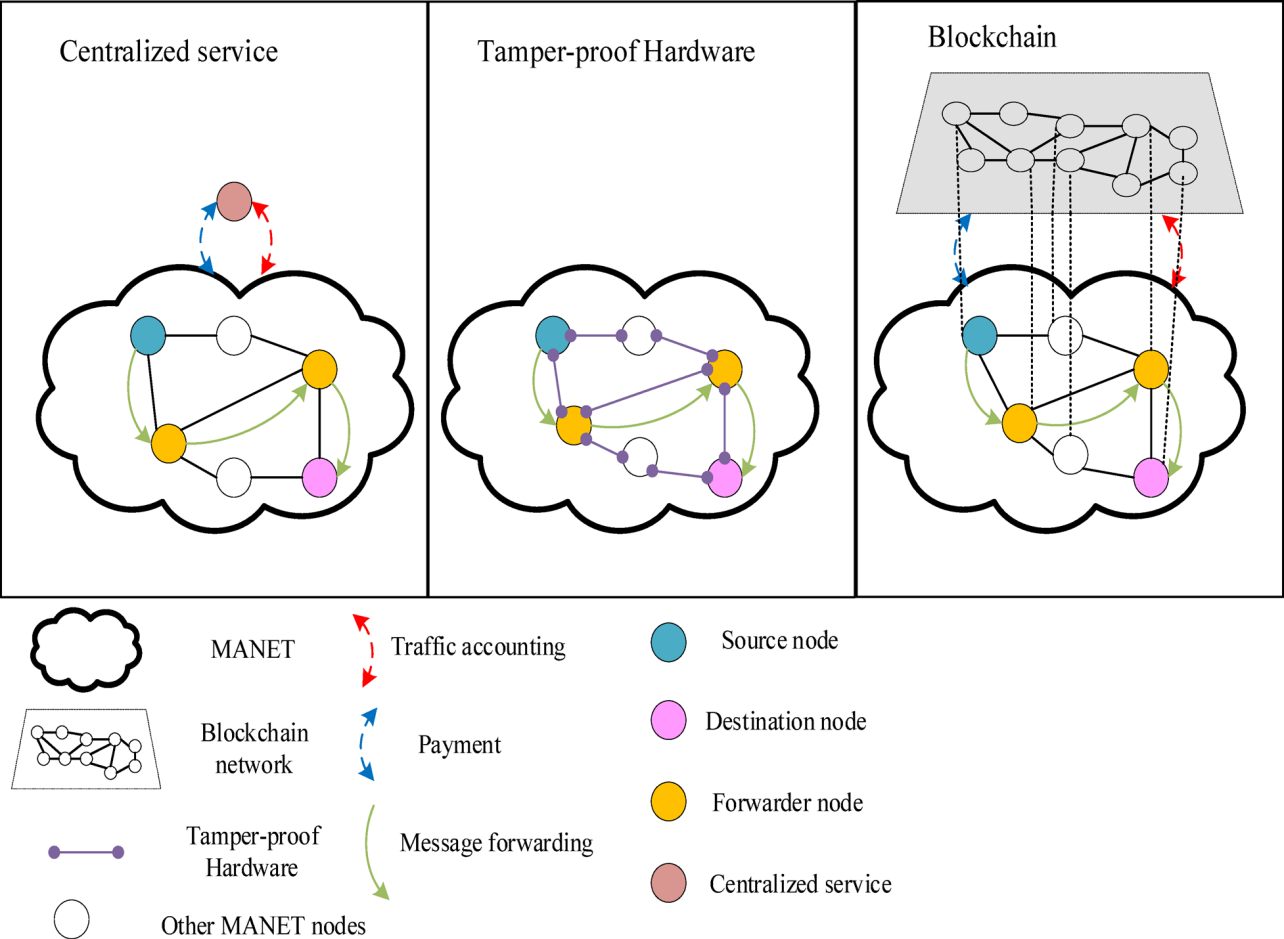
MANETs constructed using Blockchain forwarding Score of a Node.

The SBO-CNN application: Route costs field is now a part of the routing table of each node instead of hop-count field. All the neighbors are then inserted into a Reputation Table (3) holding the reputation statistics of the neighbors. Shown in Fig. 3 are these changes. Connection cost is calculated when the intermediate node receives RREQ packet and gets the reputation values of the sending node out of its Reputation Table. The destination and other intermediate nodes of SBO-CNN do not discard the RREQ packets immediately as more than one packet can be received by the destination. Owing to this fact, the final node is able to choose the shortest as well as most reliable path among the paths, traversed by the RREQ packets. Several copy RREQ packets can be forwarded to nodes thereby reducing the chances of routing loops.

In simple terms, a route P is the set of nodes that an RREQ packet has traversed. The route cost of a packet, c(P), going down path P, as calculated with the new connection metric in (4) is

4 $$c\left( P \right) = \alpha \left| P \right| + \left( {1 - \alpha } \right)\left( {\left| P \right| - \mathop \sum \limits_{{n_i} \in P} {R_i}} \right)$$

*|P|* stands for the path’s cardinality and is proportional to the hop count. After receiving many RREQ packets, the final destination node chooses the path ${P_{rcp\;}}$ that has the lowest route cost. This guarantees that, out of all the potential routes from origin to destination, the one denoted as ${P_{rcp\;}}$ is the shortest and most renowned one.

5 $${P_{rcp\;}} = argmi{n_P}c\left( P \right)$$

**Fig.4:**
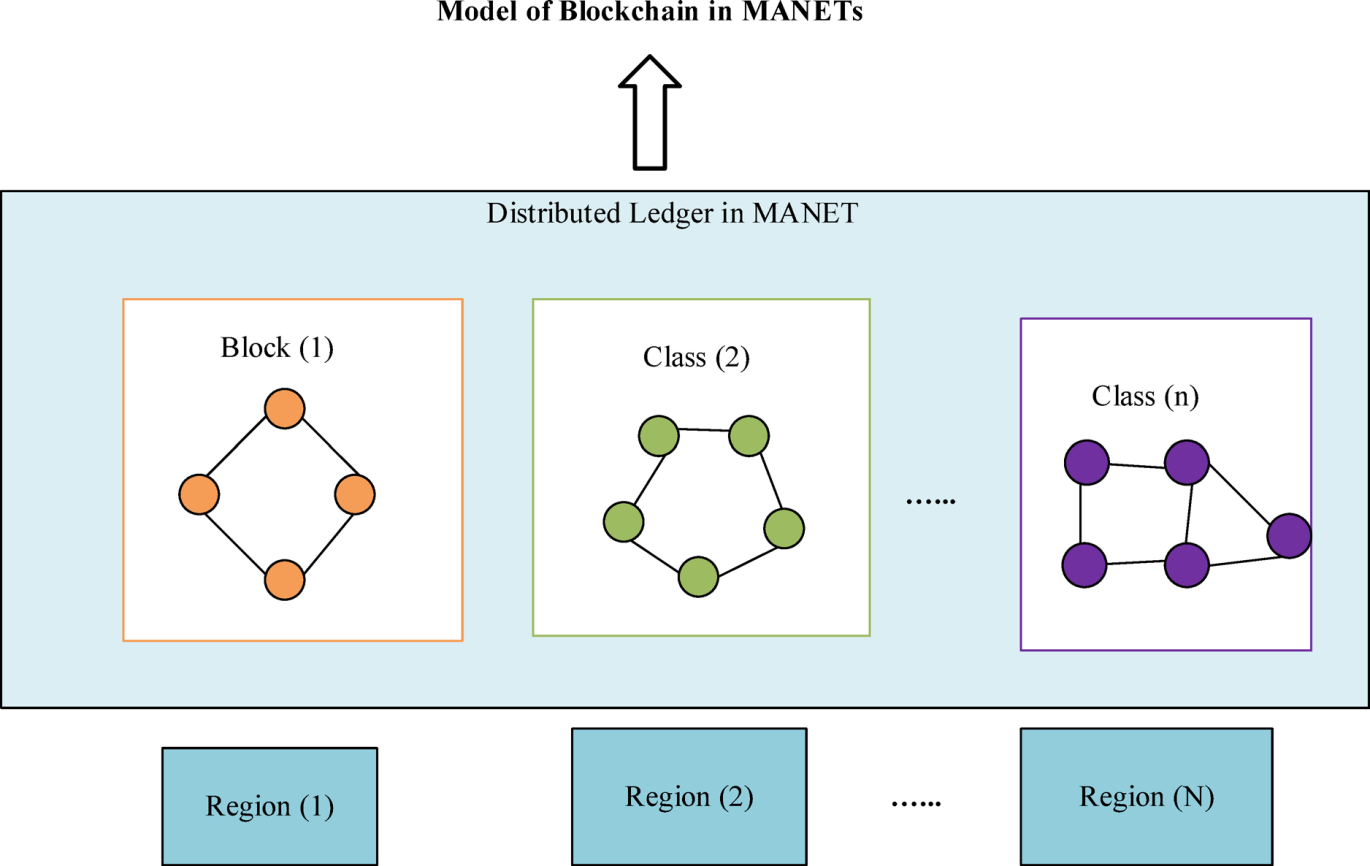
(A) proposed routing technique (b) Distributed ledger model in MANETs design of the Satin Bowerbird optimization CNN (SBO-CNN).

TThe RREP packet is sent in reverse order across the nodes in ${P_{rcp\;}}$ by the destination node. The source node takes the shortest and most reputable route, ${P_{rcp\;}}$ when it receives an RREP packet since it has the lowest route cost. Adding nodes’ reputations to a path’s route cost makes sense on paper since it encourages using more well-respected nodes and avoiding less-respected ones. Using the suggested approach, which takes node reputation into consideration in the RREQ packet’s route cost measure, guarantees the nodes’ intrinsic security. Figure 4a shows the proposed routing technique and Fig. 4b shows the Distributed Ledger Model in MANETs.

Based on the idea and mechanism of stain birds, the SBO is a novel metaheuristic algorithm. In specialized stick constructions, these birds choose females for mating. The female birds use the bowers decorated with fruit, feathers, and flowers to find the male birds to mate with. To win, the men must dance around the beautiful bowers while copying the girls’ movements and making raucous noises. The ladies seek for males who display strong passion during their frequent bower visits before choosing a mating mate. Furthermore, not every guy is successful in constructing and tending to the bowers. Since this is the case, there is a great deal of diversity in the success rates of mating. The SBO’s versatility and effectiveness in feature selection have led to its widespread use; as a result, it may achieve accuracy levels that are competitive with other methods. The SBO algorithm was developed using the following processes inspired by the stain birds’ way of life; the algorithmic design may be found in Listing 5.

1. Bowers should be generated at random.
2. Determine the price of every bower.
3. Find the most suitable bowers.
4. Although the last requirement is unmet.

- Try this formula: how likely is it that each bower:

6 $$~fit~ = \left\{ {if\left( {x_{i} } \right) \ge 0\frac{1}{{1 + i\left( x \right)}}~Else~1 + \left| {f\left( {x_{i} } \right)} \right|~\Pr ob~_{i} = \frac{{~fit~_{i} }}{{\mathop \sum \nolimits_{{n = 1}}^{N} fit_{n} }}} \right.$$

where *N*

Represents the number of bowers in the population, whereas “fit” stands for the fitness score of the $i^{th }$ solution, and $f\left( {x_{i} } \right)$ displays the fitness level of the.

metrics. What follows is the training of the CNN model by iteration over the given number of epochs. This approach calculates the probability of the bowers, which implies that the object.

To ensure full reproducibility of the proposed approach, all simulations were conducted using the NS-3 discrete-event network simulator integrated with a MATLAB-based blockchain and reputation evaluation module. The MANET environment was configured with a fixed simulation area of 100 m × 100 m, where nodes followed the Random Waypoint mobility model with uniformly distributed initial positions. Node speeds were varied between predefined minimum and maximum values to reflect realistic mobility conditions.

Each simulation scenario was executed for a duration of 900 s, during which nodes generated Constant Bit Rate (CBR) traffic with packet sizes uniformly distributed between 300 and 600 bytes. The transmission range of each node was fixed at 30 m, and IEEE 802.11 was used as the underlying MAC protocol. The number of nodes was set to 30, while the number of miners varied between 10 and 20 to evaluate the impact of mining density on ledger performance.

Blockchain operations were emulated using a Directed Acyclic Graph–based distributed ledger model. A new block was generated once the leader’s mempool accumulated 130 transactions. The cryptographic hash function SHA-256 was used for block creation, with a maximum difficulty value of 16. Each node generated transactions according to a Poisson arrival process with a rate of 0.5 transactions per second, ensuring controlled system load.

Malicious behavior was introduced by configuring a subset of nodes to perform black hole, gray hole, and false reputation injection attacks. Packet dropping probability was adjusted based on the maliciousness level assigned to each adversarial node. These settings ensure that all experimental conditions can be reproduced accurately.

### SBO-CNN training configuration

The proposed SBO-CNN model was trained using a well-defined set of hyperparameters to ensure stable convergence and optimal performance. The learning rate was initialized at 0.001, allowing efficient gradient updates while avoiding oscillations during training. A batch size of 32 was selected to balance computational efficiency and generalization capability. The model was trained for 100 epochs to ensure sufficient learning without overfitting.

The Adam optimizer was employed due to its adaptive learning rate capabilities, which improved convergence speed and stability. The Rectified Linear Unit (ReLU) activation function was used in convolutional layers to introduce non-linearity, while a sigmoid activation function was applied in the output layer for binary classification. Binary cross-entropy loss was used as the objective function to effectively measure classification error.

For the Spider Bee Optimization (SBO) component, key parameters included a population size of 30, a maximum of 50 iterations, and a fitness function based on classification accuracy and loss minimization. The SBO algorithm was used to optimize CNN weights and feature selection, enhancing overall model performance.

This combination of carefully selected hyperparameters and optimization strategy ensures that the SBO-CNN model achieves high accuracy while maintaining computational efficiency.

## Results & discussion

We put our system through its paces using NS3, a discrete event simulator. The components that make up this program may mimic many network protocols; our focus here is on those that enable the simulation of mobile mesh networks. In order to test our methods with the environment-specific protocol stack, we built a block graph app employing many NS3 modules. Here we provide the details of our two simulated cases. In the initial “No split” scenario, every node in the system travels in the same direction at the same speed. In this instance, there aren’t any network partitions. In the alternative "split subsequently to a merge" scenario, the network is first divided, and then reassembled. The primary goal of these simulations is to verify the blockgraph architecture by sustaining a DAG-based distributed ledger technology (DLT) on a mesh and mobile network; the secondary goal is to examine the effects of a network partition on the system’s operation. In order to validate the generation of blocks and see how a network split affected the creation of blocks, we monitored the amount of transaction in the leader’s mempool to back up our simulations. There is a 15-min (900-s) relative length for each simulation. To test each possibility, we modeled a network with 10 nodes. Simulation Parameters are in Table 1.

**Table 1:** Simulation parameters.

Parameters	Value/model
Routing protocol	SBO-CNN
Traffic type	Constant bit rate (CBR)
Transmission range	*30 m*
Node distribution	Uniform Distribution
Mobility model	Random Waypoint
Area	*100 m× 100 m*
Grid Size	*50 m*
Number of nodes *(N)*	30
Number of miners *(M)*	$[\mathrm{10,20}]$
Cryptographic hash function	SHA-256
Maximum difficulty $\left({D}_{max}\right)$	16
Tuning parameter *(α )* Malicious attacks modeled	0.5 Black hole attack, gray hole attack, false reputation injection attack

**Fig. 5:**
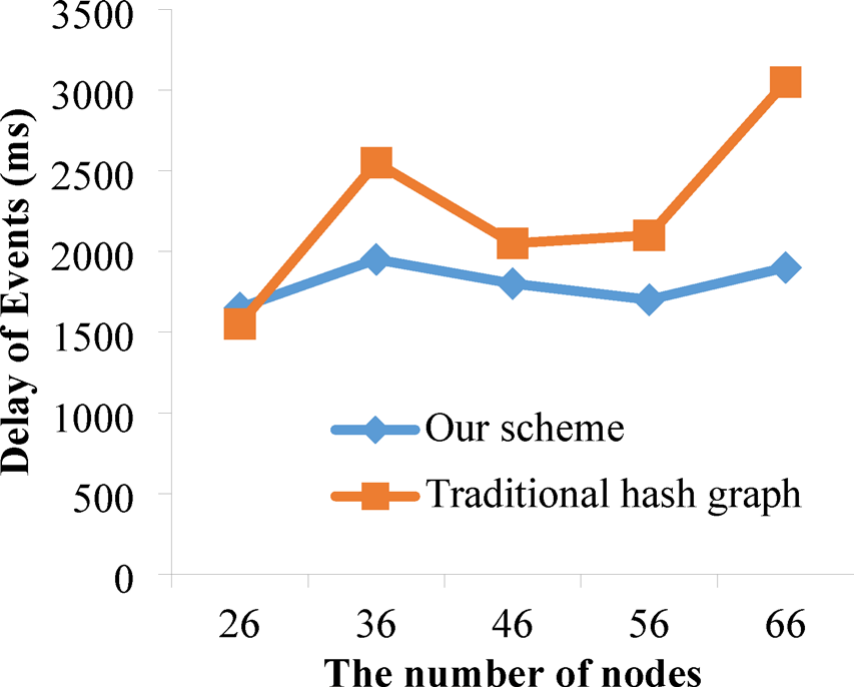
The changes of event delay with the increase of consensus nodes.

After 130 transactions are made in the mempool of the leader, the leader node will create a block. A uniformly distributed random variable is used to determine the size of a transaction. There is a 300–600 byte range for values. The average size is 58.5 KB with each block not exceeding the theoretical maximum size of a UDP packet, of 65.5 KB. Tasks of all the members of a group that is a part of the same network receive a copy of transactions created by each node. To describe the way transactions are generated at each node, we may say that each node is generating a random variable, the time between two successive generations of transactions (or the time between two arrivals), and that the random variable is of an exponential law. Next, we are able to define the coming of transactions as a Poisson process with the parameter of lambda (A). In this case, A is the arrival rate per node that can be defined as the time between two arrivals. We used a low setting of A in our first simulation to prevent overloading the system. In both cases, the rate of the arrival of the transaction is A = 0.5tx/s. Put simply, every 2 s, a transaction is generated by each node. Figure 5 displays the variations of the delay of the events as the number of consensus node increases.

Using reputation data distributed over a blockchain, we suggested SBO-CNN, a shortest, most regarded route routing algorithm. A new, trustworthy, and immutable way for trustless organizations in MANETs to reach a distributed agreement on nodes’ reputations is via the utilization of the blockchain to record node activity and the unique, diverse challenge assignment of miners inside each grid. Through the exhibit of resilience to attacks such as black holes and grey holes, we have shown how reputation of nodes can be manipulated to modify the cost measure of SBO-CNN, hence, enhancing reliability of the route selection. According to stringent simulations, the proposed approach works successfully in terms of routing performance over trustless entities.

Table 1 contains the settings used to assess SBO-CNN using a Matlab-based integrated Blockchain simulator. We think about a network with a random topology that has many nodes and miners. All of the nodes and miners have an omnidirectional antenna and a single 802.11 interface; their mobility speeds vary based on a Random Waypoint mobility model. To simulate black hole & grey hole attacks, malicious nodes are programmed to dump bursts of packets with varying probability. In order to include hostile behavior into the routing protocol’s functioning, packet dropping is solely used on data packets. This opens the door for potentially harmful nodes to be inserted in routes. We evaluate SBO-CNN and Blockchain technology in terms of their performance. The efficiency of blockchain technology: Fig. 3 demonstrates mining performance at varying numbers of miners and difficulties. The more miners in a grid, the greater the mining power or average hash rate of the pool of miners, which can generate more hashes per second. That is, a grid with a larger count of miners can produce a valid block with a bigger difficulty target at a quicker pace compared to a grid with a smaller count of miners. By holding the difficulty constant (as in Fig. 3 block mining time is reduced with the total number of miners in the grid). A valid block is produced with a difficulty of 16 after every 1.5 s with 10 miners on the same grid. The time it takes for miners on every grid to create a valid block is shown in Fig. 3. The mining strength of the miners of a grid increases directly with the number of miners, and the heterogeneous difficulty meter as (1) defines increases directly as well. Therefore, on average, an acceptable block is formed from each grid simultaneously, independent of the amount of miners. However, the most difficult block—the one that miners believe has the most credibility—and is mined in less than 1.5 s is the one that gets added to the more difficult chain.

*Assessing reputation* The reputations given to nodes, with different levels of maliciousness, are shown in Fig. 4. The likelihood of a node dropping a packet is proportional to its level of malevolence. More reputable nodes may be included in the route because the nonlinear nature of the reputation function, as illustrated in Fig. 4a, increases the reputation of mostly honest nodes and decreases the reputation of harmful nodes. Nodes that are mostly trustworthy and forward packets accurately get a reputation of 1. This means that their harmful behavior is less than 10%. Because of the large amount of reported bad transactions, malevolent nodes that consistently drop packets (malicious activities > 90%: for example, a continuous black hole assault) will eventually have a reputation close to zero. Nodes’ reputations are more easily influenced by their relatively dominating behavior if they randomly change their behavior, whether it’s dropping or forwarding packets having a particular regularity. For instance, nodes with a harmful activity level between 25 and 75% will earn a reputation that represents that level of activity. Figure 4b shows the evolution of nodes’ reputations during time. Nodes whose behavior is consistently excellent or bad tend to have their reputation values settle down faster than nodes whose behavior is inconsistent. After around ten blocks, the standing of nodes reaches a stable state within ten percent. Keep in mind that the reputation is still somewhat unaffected by the presence of a malevolent miner. Reason being, the full blockchain is used to determine a node’s repute. As the total amount of miners along with the length of the blockchain increase, the reputation is less affected by a small number of forged blocks, even if a malicious miner were to succeed in creating legitimate blocks with forged information.

**Fig. 6:**
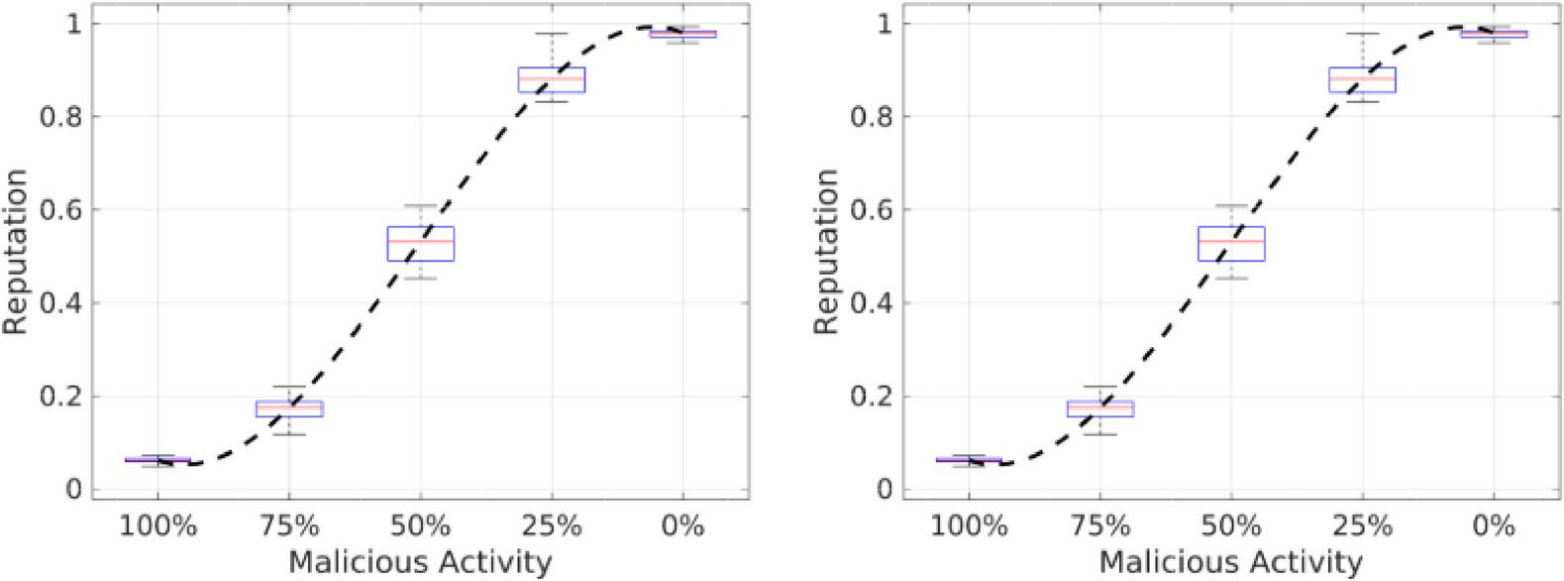
(**a**) Reputation vs maliciousness (**b**) Reputation over time.

**Fig. 7:**
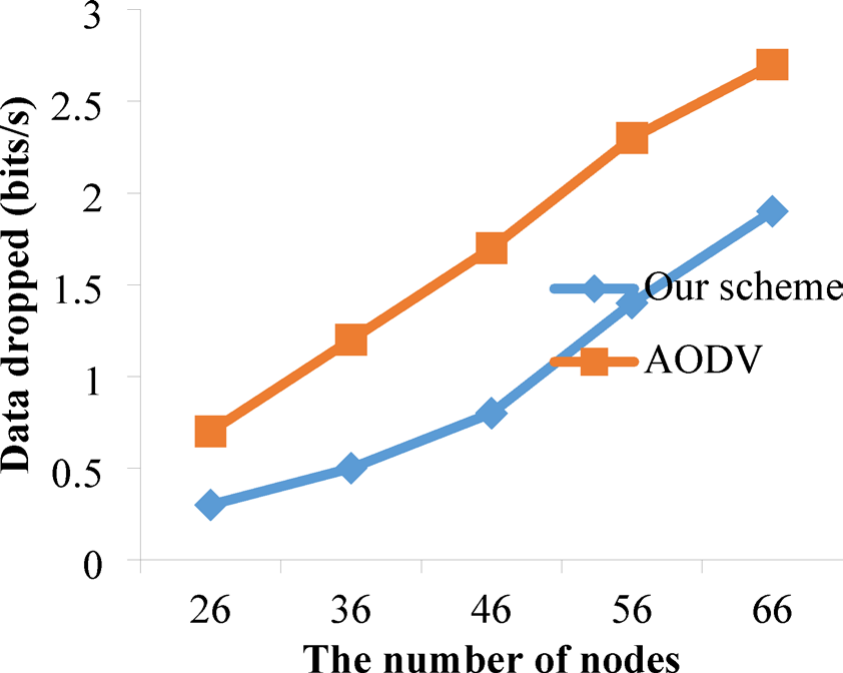
Packets dropped rate.

Routing Performance: SBO-CNN finds the shortest, most reputable route from the source to the destination node, while AODV protocol finds the most efficient route (in terms of the Hop Count). With a higher number of malicious nodes, the divergence in route selection between the two methods becomes more apparent. This is because keeping harmful nodes out of the route is one of SBO-CNN’s primary goals. The frequency with which SBO-CNN deviates from AODV’s route, together with the percentage of malevolent nodes, is shown in Fig. 6a. The number of pathways chosen by SBO-CNN that deviate from AODV grows as the ratio of malicious nodes increases. Packets dropped rate are shown as graph in Fig. 7.

The measurement for packet loss is given by the equation ∎ Dropped Packets | =| Packets Sent |-| Received Packets ∎, where |⋅| denotes the quantity of the specific packet type. When ten percent of the nodes exhibit a black hole attack (losing all their data packets), Fig. 6b examines the number of packet losses in AODV and SBO-CNN. Since evaluating the reputation and behavior of nodes takes time, SBO-CNN initially performs similarly to AODV (Fig. 4 b). Nevertheless, SBO-CNN has much fewer packet losses than AODV after the nodes’ reputations have been evaluated. This occurs because SBO-CNN may utilize node reputation to detect rogue nodes and redirect packets to a more trustworthy channel, therefore avoiding these nodes. Since the path selected by (6) compromises between the total reputation of the nodes throughout the route and the total amount of hops to the destination, there was no zero number of packets discarded by SBO-CNN. To further decrease packet loss, use a lower α and prioritize the reputation cost above the hop count in (5). On average, SBO-CNN reduces the packet loss rate compared to AODV by around 12%. Tables 2, 3, 4, 5 and 6 shows the comparative analysis with different metrices.

**Table 2:** Comparative analysis of accuracy.

Number of packets	AODV	CNN	SBM-CNN
100	61	72	88
200	74	75	83
300	77	74	81
400	71	81	93
500	81	94	97

**Table 3:** Comparative analysis of F-score.

Number of packets	AODV	CNN	SBM-CNN
100	65	62	71
200	67	75	77
300	73	71	72
400	71	72	88
500	86	80	83

**Table 4:** Comparative analysis of precision.

Number of packets	AODV	CNN	SBM-CNN
100	72	85	81
200	76	86	84
300	83	87	83
400	85	89	95
500	87	90	93

**Table 5:** Comparison of recall.

Number of packets	AODV	CNN	SBM-CNN
100	57	65	64
200	58	68	67
300	62	69	79
400	65	73	72
500	67	79	92

**Table 6:** Comparative analysis of RMSE.

Number of packets	AODV	CNN	SBM-CNN
100	61	61	52
200	66	57	57
300	68	58	58
400	55	61	54
500	51	42	42

To improve the reliability of the reported results, each simulation scenario was executed independently 20 times using different random seeds. The final performance metrics, including packet delivery ratio, packet loss rate, end-to-end delay, and reputation convergence, were obtained by averaging the results across all runs.

For each metric, 95% confidence intervals were computed to quantify variability and statistical reliability. Where applicable, paired t-tests were conducted to evaluate the statistical significance of performance differences between the proposed SBO-CNN approach and the baseline AODV protocol. Results demonstrating a p-value less than 0.05 were considered statistically significant.

These statistical measures confirm that the observed improvements are consistent and not due to random variation in node mobility or traffic patterns.

## Conclusion

While the proposed blockchain-assisted SBO-CNN routing framework demonstrates improved robustness against malicious behavior and enhanced routing reliability, it is important to note that all findings are based on simulation-based evaluations. Although the results indicate promising scalability and trust convergence characteristics under controlled conditions, real-world deployments may introduce additional constraints such as hardware limitations, unpredictable mobility, and network heterogeneity.

Therefore, the scalability and trust guarantees presented in this work should be interpreted within the context of simulated MANET environments. Future work will focus on large-scale simulations and real-world testbed implementations to further validate the proposed approach under practical deployment scenarios.

## Data Availability

The datasets used and/or analysed during the current study available from the corresponding author on reasonable request.
